# A comprehensive genomic meta-analysis identifies confirmatory role of *OBSCN* gene in breast tumorigenesis

**DOI:** 10.18632/oncotarget.20404

**Published:** 2017-08-23

**Authors:** Barani Kumar Rajendran, Chu-Xia Deng

**Affiliations:** ^1^ Cancer Research Centre, Faculty of Health Sciences, University of Macau, Macau SAR, China

**Keywords:** breast cancer, OBSCN gene, OBSCURIN, cell adhesion, EMT

## Abstract

The giant multifunctional protein “OBSCURIN” is encoded by *OBSCN* gene and is mostly expressed in cardiac and other skeletal muscles responsible for myofibrils organization. Loss of OBSCURIN affects the entire downstream pathway proteins vital for various cellular functions including cell integration and cell adhesion. The *OBSCN* gene mutations are more frequently observed in various muscular diseases, and cancers. Nevertheless, the direct role of *OBSCN* in tumorigenesis remains elusive. Interestingly, in clinical breast cancer samples a significant number of function changing mutations have been identified in *OBSCN* gene. In this study, we identified a significant role of *OBSCN* by conducting an integrative analysis of copy number alterations, functional mutations, gene methylation and expression data from various BRCA cancer projects data available on cBioPortal and TCGA firebrowse portal. Finally, we carried out genetic network analysis, which revealed that *OBSCN* gene plays a significant role in GPCR, RAS, p75 or Wnt signaling pathways. Similarly, *OBSCN* gene interacts with many cancer-associated genes involved in breast tumorigenesis. The *OBSCN* gene probably regulates breast cancer progression and metastasis and the prognostic molecular signatures such as copy number alterations and gene expression of *OBSCN* may serve as a tool to identify breast tumorigenesis and metastasis.

## INTRODUCTION

Cancer in humans is a huge health burden in modern era worldwide. In recent years cancer genomics is assumed as a latest advancement in cancer research, which starts from disease identification and leads to personalized therapy. Owing to this scientific advancement, several key genetic elements have been identified and characterized in various cancer types. In human carcinogenesis events, cells lose their adhesion, integrity and others morphological characteristics and gain the invasive and migratory properties leading to cellular transitions which are called epithelial-to-mesenchymal transition (EMT), which is the most crucial step in initiating cancer metastasis [1, 2]. Large number of genes and their corresponding proteins are involved in EMT associated pathway including OBSCURIN. The giant OBSCURIN is encoded by unique gene called *OBSCN* located on chromosome 1 at loci q42 [3]. The giant OBSCURIN has two isoforms namely OBSCURIN-A & B [4–6]. The OBSCURIN-A comprises of immunoglobulin (Ig) and fibronectin type-III domains located in the amino terminal while in carboxyl terminal contains several signaling domains including Isoleucin & Glutamine calmodulin-binding (IQ), SRC homology-3 (SH3) domains, pleckstrin homology (PH) and a Rho-guanine nucleotide exchange factor (Rho-GEF) domains which are scattered in the non-modular sequences. The OBSCURIN-B or myosin light chain kinase (MLCK) isoform contains two serine/threonine kinase (STK) domains, which replace the non-modular carboxyl terminus of OBSCURIN-A. Additional to these domains, ERK kinase domain exists for phosphorylation along with two Ig domains [3, 4, 7, 8]. Every OBSCURIN domain regulates vast number of cellular and functional roles such as cell adhesion, migration and cell morphology, etc. Apart from these functional roles, OBSCURIN-A/B are also involved in cellular co-ordination that prevents cells from going in an EMT process [4, 9]. OBSCURIN MLCK family domains chiefly maintain the cellular organizations and contractility [10]. Moreover, a huge number of copy number alterations and mutations were observed in *OBSCN* gene in many cancer types, although, *OBSCN* is highly mutated in breast cancer. The main study is to uncover the functional mutations and the active role of *OBSCN* in breast tumorigenesis. A few studies on this gene also proved that, the reduced or altered *OBSCN* gene expression largely disturbs the cellular integration and activates cancer initiation; therefore, *OBSCN* may function as a tumor suppressor gene and prevent cellular transition [11, 12]. *OBSCN* gene is mostly expressed in many cancer types although the real functional association in cancer is still uncertain. Several studies on *OBSCN* gene mutations revealed potential roles of OBSCURIN in melanoma, glioblastoma, colorectal, lung, breast and pancreatic cancer [13–15]. Also, our recent study on breast cancer driver mutations genes also emphasized *OBSCN* as one among the 63 top candidate driver genes [16]. Here we will mainly focus on copy number variation, non-silent mutations, promotor methylation followed by epigenetic changes and their consequences in breast cancer formation.

### Mutations and expression of *OBSCN*: genetic predisposition to tumorigenesis

Recent advances in high throughput sequencing support genomics in a magnificent way. In every cancer, vast numbers of genes are mutated, therefore the identification of real genetic elements responsible for carcinogenesis becomes quiet challenging. *OBSCN* gene is frequently and consistently mutated in various cancers with a strong correlation with breast, colorectal and other female related cancers. Recent studies revealed that TP53 and *OBSCN* genes are highly mutated among 189 candidate genes in breast and colorectal cancers [13, 17]. However, scientific background on *OBSCN* mutation and its impacts are limited in comparison to the other key genes involved in breast cancer [18, 19]. Mutations in *OBSCN* gene (> 15%) are observed in breast cancer patient samples published by TCGA. Furthermore, a recent study on *OBSCN* gene in breast cancer revealed that the loss of giant OBSCURIN protein increases the cell migration with more metastatic characteristics [20].

However, recent studies revealed that OBSCURIN expression loss causes functional abnormality, which increases the probability of cancer in human breast epithelial cells. Perry et al. (2012) found that knocking down *OBSCN* significantly affected breast epithelial cells in both growth and biological property, such as cellular adhesions, cell-cell communications, etc. As a result, expression level of other interlinked proteins such as E-cadherin, α and β-catenin involved in cell-cell junctions were largely affected. Meanwhile, an elevated level of p120-catenin prevents the cell adhesion and stimulates mesenchymal proteins, which stimulate cellular morphogenesis leading to metastasis [21–23]. The actin and microtubule cytoskeleton dysregulation is responsible for cellular transformation and migration found in many cancers and activation and deactivation of Rho-GTPase family proteins majorly regulates actin filaments through Ras homolog gene family, member A (RHOA) signaling. The loss of *OBSCN* caused RHOA signaling impairment leading to breast cancer initiation, progression and metastasis. [12, 24–28]. The consequent event of *OBSCN* loss may also affect tubulin microtentacles (McTN) formation. McTN is the key process in metastatic breast cancer and it increases metastatic probability and endothelial coupling followed by circulating tumor cells (CTC) [29–31].

Similarly, low levels of epithelial proteins accelerate phenotypic changes in cells, which further affect focal adhesion and increase of F-actin dynamics in cell-cell contacts [28]. Another interesting study proved that, an active oncogene, *K-RAS* in the *OBSCN* knockdown cells increases tumorigenesis probability [9]. The *OBSCN* down regulation also affected RHOA-mediated pathways leading to dramatic reduction of Rho activated kinase (ROCK) and its targeting proteins such as myosin light chain (MLC), lim kinase (LimK) and cofilin [4, 8, 22, 29, 30]. In this study, we reviewed and analyzed *OBSCN* gene and its association with breast cancer. The high throughput sequencing data for breast cancer mutations in clinical samples were retrieved from various breast cancer projects available in cBioPortal (www.cbioportal.org), Pan-Cancer (https://www.synapse.org), mRNAseq and Methylation data were retrieved from Firebrowse (http://firebrowse.org/) data portal, which provides multi-tier cancer analysis. The analyses of the overall mutation rates and the mutational patterns of *OBSCN* gene, as well as their impact on downstream pathways may help us to predict feasible drug targets for breast cancer.

### The *OBSCN* mutations in human cancers using high throughput analysis

**Figure 1:**
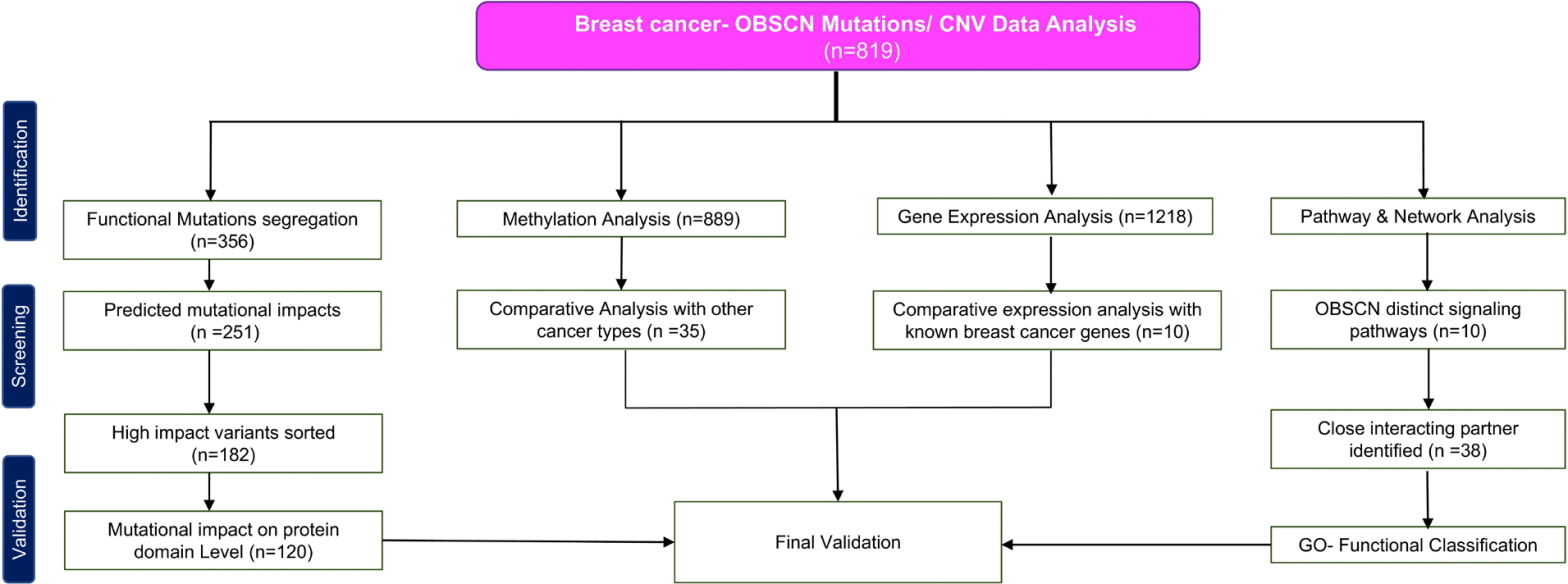
Flow diagram summarizing selection and validation of the present meta-analysis on OBSCN gene

**Figure 2:**
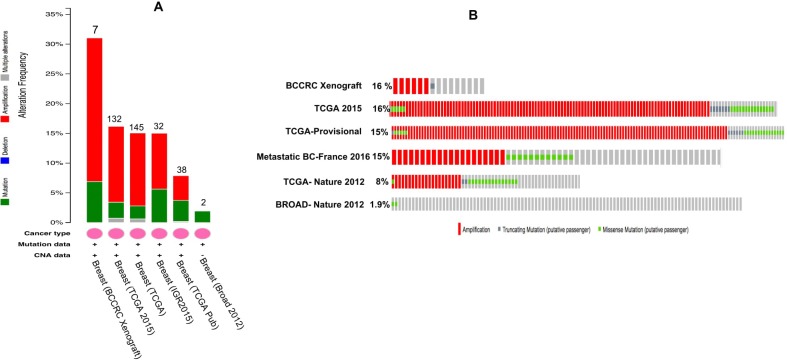
Overall *OBSCN* gene mutations across various breast cancer projects data of cBioportal (**A**) number of *OBSCN* mutations/copy number variations found in each project; (**B**) Distribution of *OBSCN* gene mutations across the patient samples (%).

**Figure 3:**
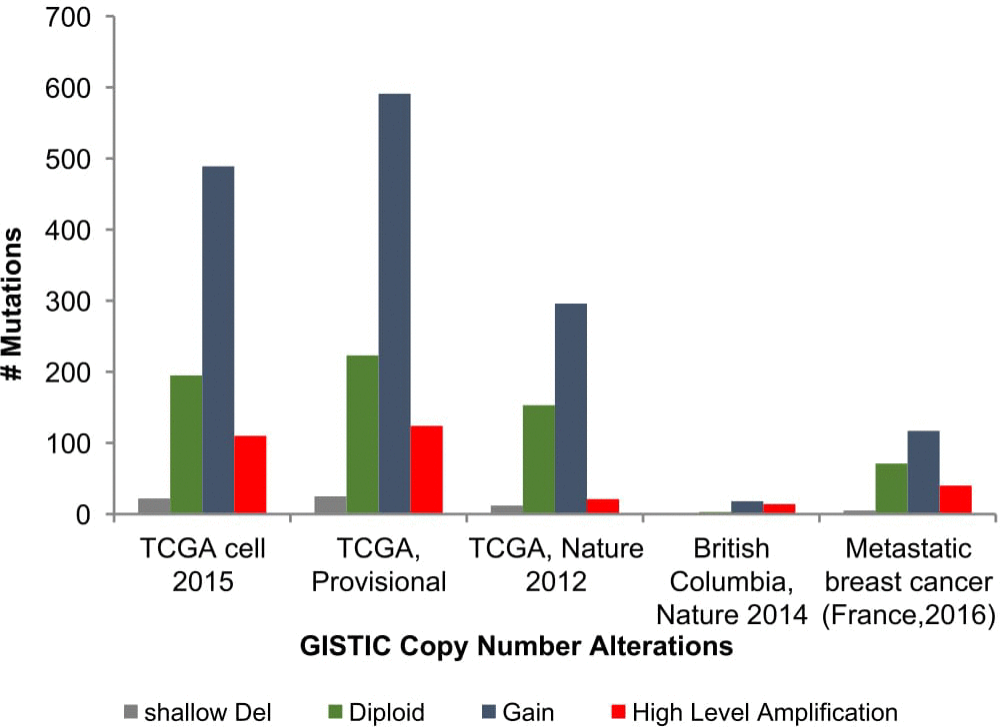
GISTIC copy number analyses of *OBSCN* gene in various breast cancer projects found with more number of gain and amplification mutations

Apart from breast cancer, *OBSCN* gene is widely mutated in many other cancer types as well (Supplementary Figure 1) (www.cbioportal.org). Among the various cancer types breast cancer cases are observed with significant numbers of *OBSCN* mutations (*n* = 819) with average mutation frequency of 18%. The overview of this work and study selection are given in the Figure 1. The overall mutations percentage of OBSCN gene in various breast cancer projects are illustrated in Figure 2 (www.cbioportal.org). The large numbers of functional mutations were observed and *OBSCN* copy number analyses were performed using GISTIC program and many copy gain, amplification and few deletion mutations were observed in major breast cancer projects data (Figure 3). From the overall DNA copy number analysis, we found that *OBSCN* gene had more number of copy gain and amplification mutations and these variations may potentially affect the giant OBSCURINs protein level and their subsequent expression.

**Figure 4:**
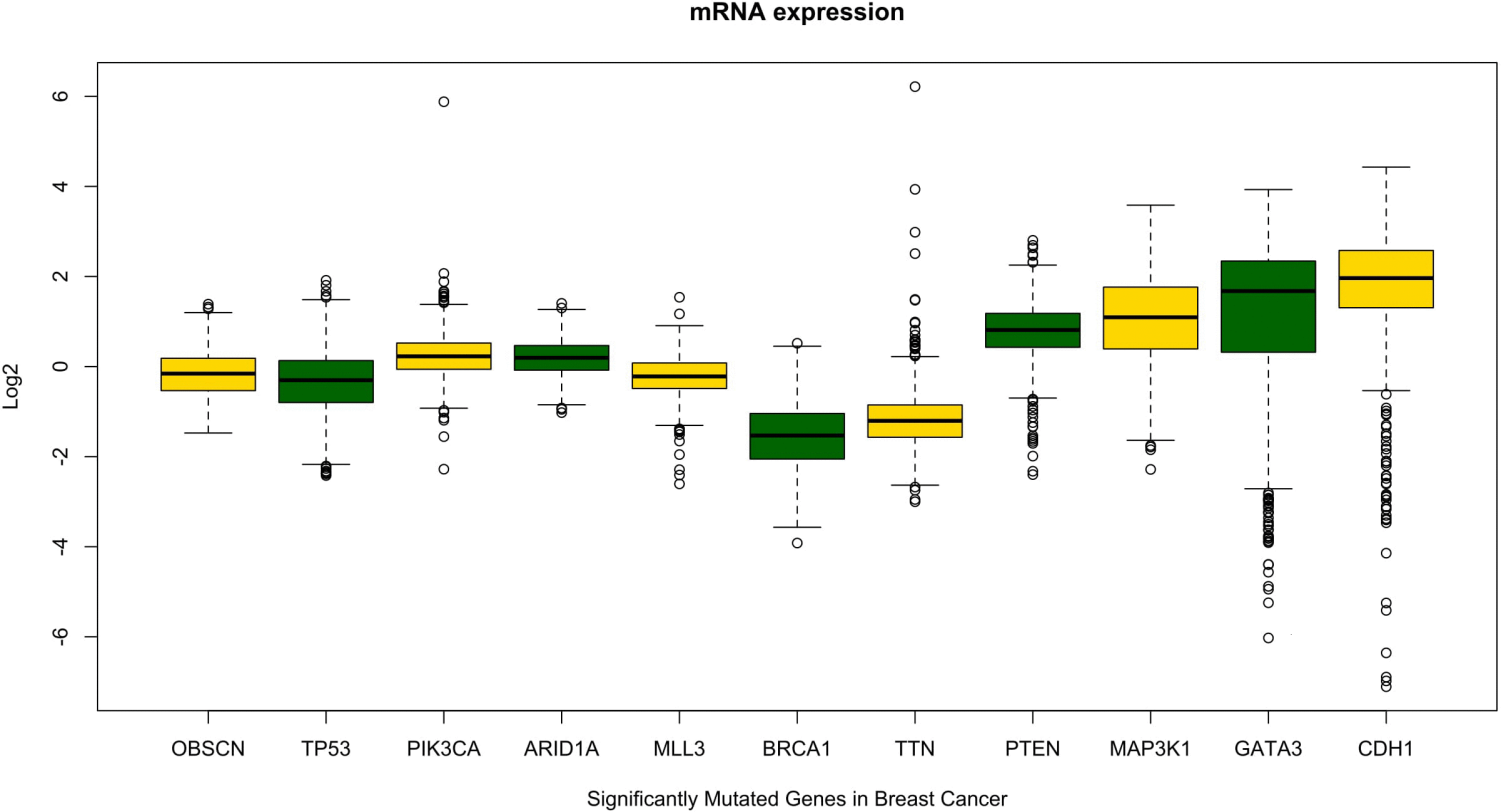
Comparative evaluation of *OBSCN* gene expression pattern with other known breast cancer genes shows *OBSCN* gene has similar expression pattern with some of the known breast cancer driver genes

**Figure 5:**
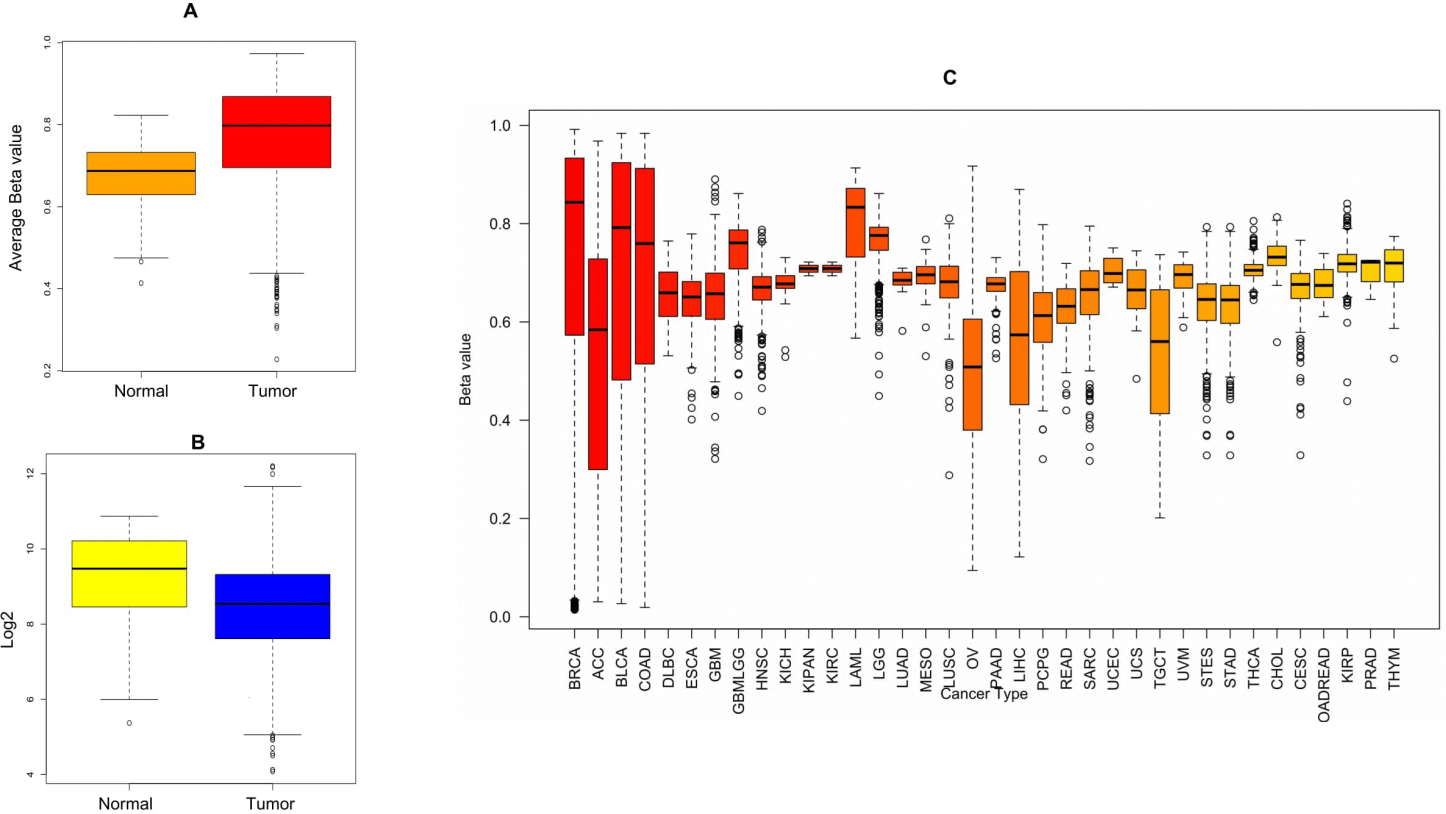
(**A**) *OBSCN* methylation profiles of normal (*n* = 98) and breast tumor samples (*n* = 720) comparison; (**B**) Gene expression comparison between normal and tumor samples; (**C**) Comparison of methylation profiles showed that *OBSCN* gene largely hypermethylated in breast cancer than other cancer types.

The significant copy number alterations are observed and their outcomes are predicted using GISTIC copy number analysis [32]. The overall mutation profiles of *OBSCN* gene in breast cancer shallow loss, diploid, gain and high level amplifications are classified based on distinctive copy number profiles and their clinical settings [33, 34]. This analysis also revealed a huge number of gain and high level amplification mutations in *OBSCN* gene found in invasive ductal carcinoma (IDC), followed by invasive lobular carcinoma (ILC) and so on. These results indicated that *OBSCN* gene might play an active role in invasion of cancer cells to distant organs and other metastatic processes. The subsequent mRNA expression analysis was performed using breast cancer patient data and based on the median Log2 value, the *OBSCN* gene is seen down regulated in majority of the mutated TCGA data. To confirm the *OBSCN* gene expression we also compared with other well-known breast cancer genes such as *TP53, PIK3CA, ARID1A, BRCA1*, etc. and found that these genes had closer expression levels and it indicated that *OBSCN* gene mutations may also have a positive correlation with breast cancer dispositions (Figure 4) (https://cran.r-project.org/); (www.cbioportal.org); (https://cancergenome.nih.gov/). In addition to that, *OBSCN* methylation profiles were also assessed using breast cancer methylation data by calculating beta value and found *OBSCN* gene apparently hyper-methylated. Then we compared methylation profiles of *OBSCN* gene over various cancer types and found the gene is hyper-methylated in breast cancer data, which indicates its methylation may also play an important role in disease etiology. We used promotor methylated and gene expression samples of *OBSCN* gene from TCGA wanderer (http://maplab.imppc.org/wanderer/). The *OBSCN* methylation and gene expression was compared with normal (*n* = 98) and tumor (*n* = 720) samples at cg09411356 and interestingly, the hyper methylation on *OBSCN* gene significantly reduced gene expression in tumor than the normal samples. The detailed methylation profile of single probe and its corresponding gene expression and comprehensive comparison of methylation pattern over major cancer types is depicted in Figure 5. (https://xenabrowser.net/#). The downregulation of *OBSCN* may affect cell-cell adhesion of breast epithelial cells and cells undergo EMT process [12]. Further we analyzed the expression of *OBSCN* gene in various immunohistochemical breast cancer molecular subtypes. Nearly 73% cases found with luminal A (46.99%) and luminal B (26.05%) type of disease followed by other subtypes including basal (10.7%), Her2(8.9%), normal (3.78%) and claudin-low (3.56%). These molecular subtypes classifications distinctly emphasized, *OBSCN* is vital gene may involve in the cell proliferation. Moreover, several previous studies on *OBSCN* gene mutations and its association with other cancers were identified by intensive literature search and the detailed information such as cancer types, mutation profiles, co-mutating genes, amino acid variations and supporting citations are tabulated (Table 1).

**Table 1:** The *OBSCN* gene mutations and their associations with various cancer types

Disease Types	Most common co-mutation with OBSCN gene	Major mutations type	Specific Amino acid variations	References
Breast Cancer	*TP53*	Missense	NA	[13, 21, 35]
Lung Cancer; Respiratory Disease	*TP53, ARID1A, PTEN, KRAS, MYC, PIK3CA, BRAF, EGFR, NRAS*	Missense	NA	[36, 37]
Gastrointestinal Stromal tumor and Leiomyosarcoma	*C9orf65, TTN, SPEG*	Missense	NA	[15, 38, 39]
Colorectal cancer	*TP53*	Missense	NA	[13, 35]
Prostate cancer	*CLDN7, STRA13, FLNA FAM83H, CLDN7, ARFGAP3, KDM2A,*	Missense	NA	[40, 41]
Melanoma	*EPHA3, TTN*	Somatic Missense	E4574K	[14]
Cervical/ Endometrial and Ovarian cancer	*Tp53, PIK3CA, FBXW7, NEB, DNAH11, ORAI2, RNF19B, SPTA1, UBA2, UTRN, BSN*	Missense	NA	[42]
Liver & Pancreatic cancer	*ATPAF1, TRPM4, MLL3, ARID1A, ARID1B, ARID2, SOS1, MROH1*	Missense	NA	[14, 19, 43, 44]
Glioblastoma	*TTN*	Germline Missense	R4558H	[13, 14]
Esophageal Cancer	*C9orf65, TP53, ARID1A, MUC17*	Missense	NA	[45]
Sarcoma	*C9orf65, PRUNE2*	Missense	NA	[15]
Nephroblastoma and other kidney cancers	*PTHB1*	Missense	NA	[46, 47]
Cardiomyopathy	*TTN, MYH7, DSP, VCL, LAMA4, MYOM1, TNNC1, TNNI3*	Truncating Mutation	NA	[47–51]

### Functional analysis of differentially expressed *OBSCN* gene in breast cancer

Mutations in the *OBSCN* gene largely affect various interlinked proteins involved in cell adhesion and integration process [52]. Functional mutations in *OBSCN* largely affects E-cadherin, α-catenin and β-catenin expressions which initiate epithelial cells dispersions and subsequently increase the level of F-actin followed by cell migration [20, 53–55]. Actin filaments are vital for cellular mechanistic amoeboid movements accelerated by filapodia or pseudopodia like structures formed in cytoplasmic regions [56, 57]. A recent study showed that *OBSCN* functional gene mutations are responsible for the loss of vital protein expressions [21]. The loss of *OBSCN* appears to be consequent event in primary to metastatic breast cancer majorly affecting cellular integration. The protein kinases (PKs) are from the larger protein family involved in various cellular, structural and functional mechanisms in humans. The Serine/Threonine Kinase (STK) is one of the important protein kinase involved in cellular cytoskeletal and integration roles such as cell proliferation, differentiation and apoptotic process, etc. [58, 59]. The STK actively binds with p120-catenin and regulates cadherin facilitated intercellular adhesions and E-cadherin-p120 bound complex also plays a crucial role in maintaining the equilibrium of cadherin levels [60, 61]. The *OBSCN* mutations affect E-cadherin level, lead to flux in adherens junctions and increase cell disintegration followed by cell movement [62]. The *OBSCN* gene mutations heavily affect STK protein activity and also act as a ligand for small ankyrin1 (sANK1). sANK1 are integral proteins to the fundamental spectrin-actin cytoskeleton involved in cellular movement activation and proliferation, which cause metastasis [63]. Moreover, *OBSCN* gene also acts as a kinome regulatory element that regulates few other kinase family proteins in either direct or indirect manner.

### Gene interaction analysis revealed that *OBSCN* gene is positively correlated with protein binding and cell transition associated genes

**Figure 6:**
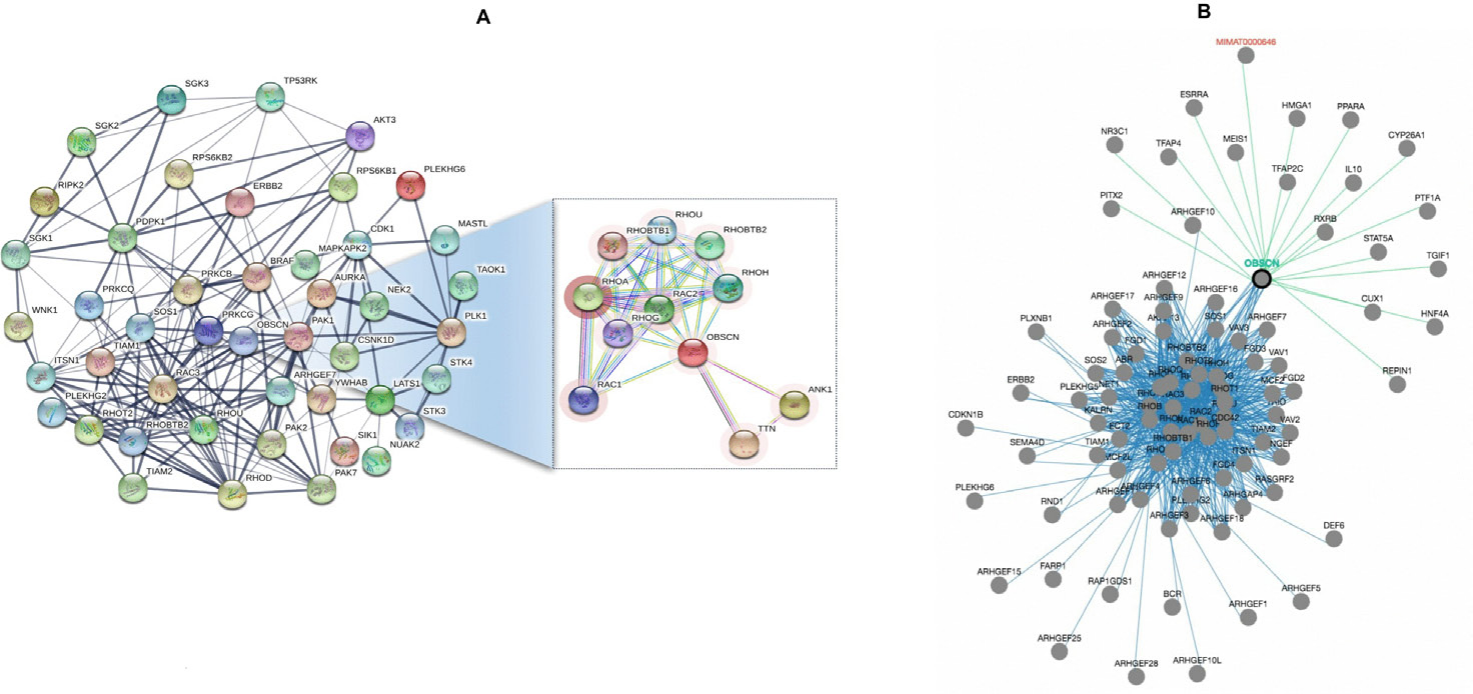
(**A**) Mutation specific *OBSCN* functional association network and its interacting network partners identified using STRING database; (**B**) Gene specific interactions pathways were identified using pathway commons network visualizer and many interacting partners of *OBSCN* are cancer-associated genes.

The *OBSCN* gene mutations have significant relationship with genes responsible for cellular structural and functional roles. Also, the expression of *OBSCN* gene is positively correlated with few other genes such as *TOP1MT, MYC, TARPBP1, ADCK3, TRIM17*, and *RND2*. Initially we used metastatic breast cancer data (Metastatic Breast cancer-France 2016) for network analysis and revealed many participating genes such as *RHOT2, RAC3, RHOD, ARHGEF7, PAK1, PDPK1, AKT3*, *RIPK2, ERBB2, PRKD1*, etc. which are positively correlated with amplification mutations and have strong association with cell proliferation, differentiation, tumorigenesis and other metastasis features. Similarly, we used British Columbia Xenografts (British Columbia, Nature 2014) data linked with many interesting genes such as *RHOB, ITSN1, RHOD, ERBB2, AKT3, BRAF, CDK1, MASTL* along with eight more genes which were highly correlated with cell signaling and cell migration functions. The breast invasive carcinoma (TCGA, Provisional) data showed that the *OBSCN* had strong interaction with *PAK1* gene through *RHOU* and *RAC3* genes. The breast cancer oncogene, *PAK1,* acts as a hub gene connecting two sub networks. In first sub network, *PDPK1* gene a central gene linked with other significant genes such as *AKT3, ERBB2, SGK3, PRKCB, PRKCQ,* etc. and the second sub network is connected with *STK3, AURKA, CSNK1D, NEK2*, etc. The PAK1 and PDPK1 genes are strongly associated with breast tumorigenesis and cell migration in metastatic event [64, 65]. Along with the afore-mentioned genes, *OBSCN* is interconnected with other genes such as *PAK2, AKT1, TP53RK, NEK2, WNK1, CSNK1D*, etc. which are frequently mutated in invasive breast carcinoma (Nature 2012) (Cell 2015). The detailed *OBSCN* mutation specific network neighbors were identified using functional protein association network using STRING (http://string-db.org/). Additional pathway specific networks were also used to identify and validate the genes involved in *OBSCN* associated pathway using PCViz (http://www.pathwaycommons.org/) and detailed networks are illustrated in Figure 6.

### *OBSCN* genetic alterations lead to cell survival and switches towards EMT

**Figure 7:**
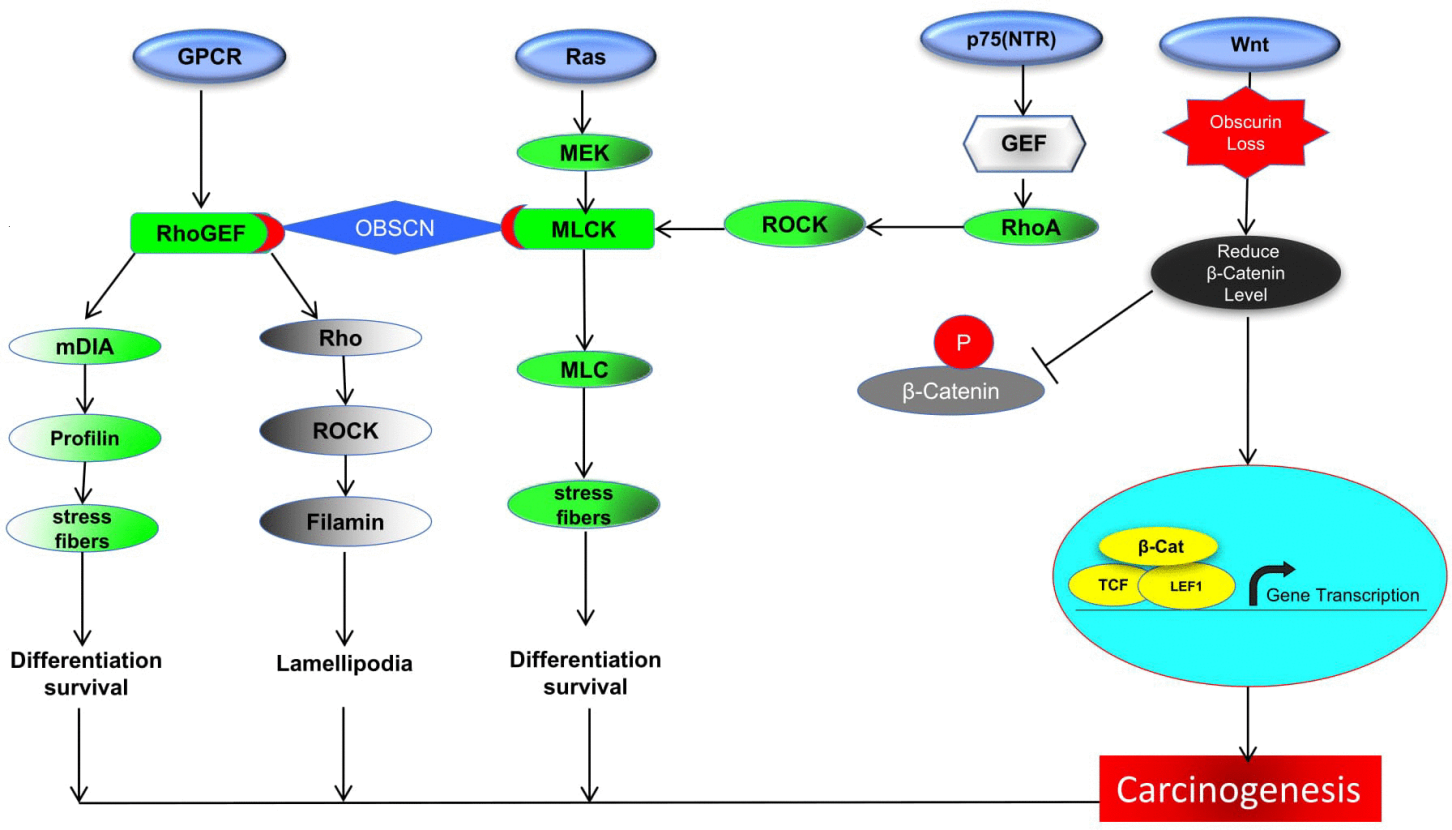
The *OBSCN* gene involved either direct or indirect pathway of GPCR, Ras, p75 or Wnt signaling (In GPCR pathway, *OBSCN*-RhoGEF is simultaneously regulates mDIA, Rho and their downstream genes which are involved in cell differentiation and lamellipodia formation; the other chain of *OBSCN*, MLCK and its downstream pathway activates MLC leads to formation of stress fiber which cause cell differentiation and survival; On the other way RhoA activated from p75 (NTR) pathway also involved in Ras pathway regulates down regulates MLCK; in Wnt signaling *OBSCN* disequilibrium leads to β-catenin loss which leads to carcinogenesis.

**Figure 8:**
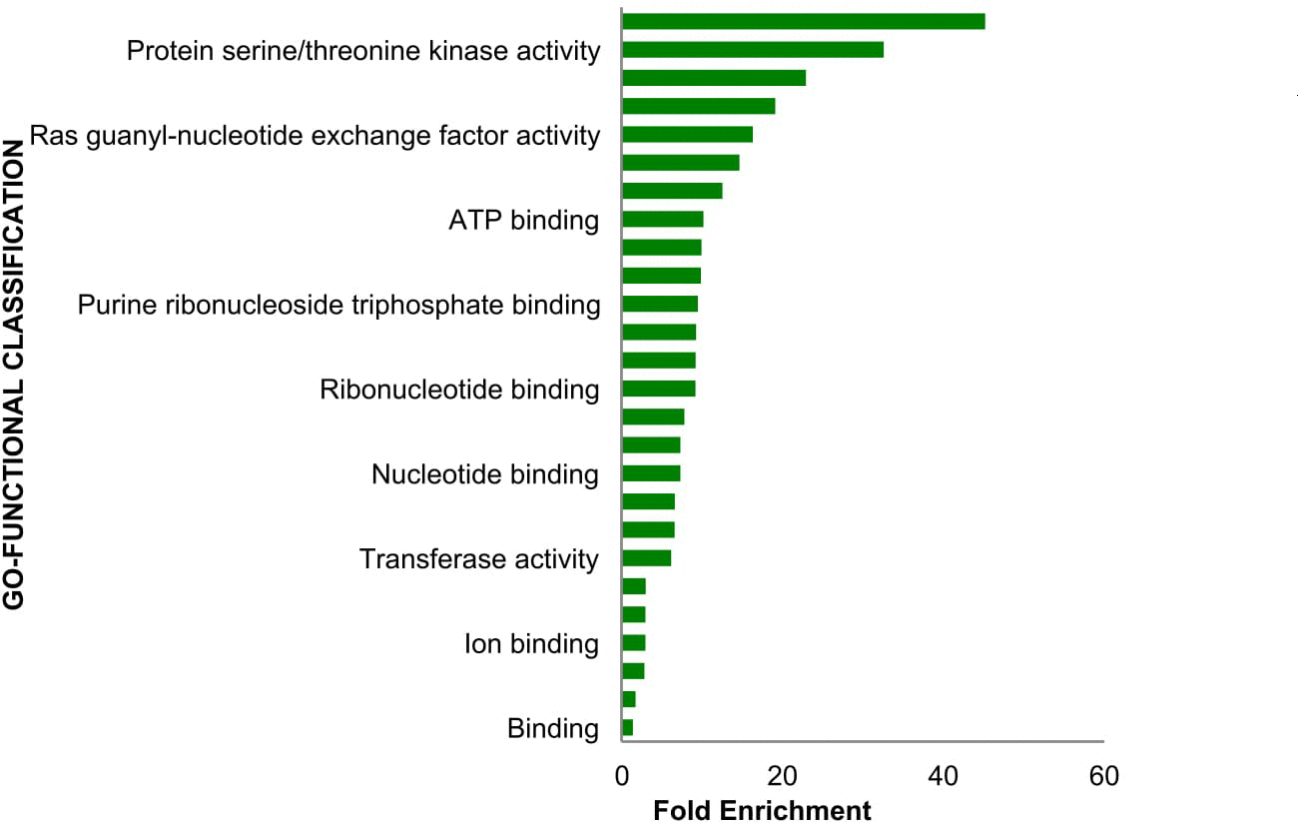
Molecular functional classification analysis of *OBSCN* gene and its network neighbor genes showed that *OBSCN* gene majorly involved in binding activity with various molecules

Substantial evidences propose that *OBSCN* functional mutations may have strong associations with Wnt signaling regulated by a series of activators along with β-catenin [66]. In the nucleus, β-catenin binds to transcription activation elements (TCF/LEF), leading to the activation of the target genes (specifically *CCND1*, *c-MYC*, and *FN1*) responsible for the regulation of cell proliferation and differentiation [67–69]. Mutational event of *OBSCN* gene majorly affects β-catenin downstream signaling leading to lower down or over expression of genes such as *CCND1*, *c-MYC*, and *FN1*, which stimulates several types of cancers including breast, ovarian, lung, pancreatic, colorectal, and uterine carcinoma [70–75]. The elevated level of fibronectin or loss of proteins such as *E-CADHERIN*, Β-CATENIN, etc. may cause cellular proliferation followed by EMT, which render patients overall survival. Henceforth, *OBSCN* gene is believed to be an indirect regulator of fibronectin expression in breast and colon cancer [72, 76]. The fibronectin malfunction may also affect vital proteins such as integrin and other transmembrane receptors responsible for cell-cell contact, extracellular matrix (ECM) communications, cell adhesion and accommodate several ligands such as collagen, laminin and vitronectin. Fibronectin is important protein which activates integrin and is involved in the regulation of cellular signal transduction, cell proliferation, gene expression and cytoskeletal reorganization [77]. Hence, the *OBSCN* gene mutation perhaps affects the regulation of β-catenin and other regulatory elements in Wnt signaling pathway largely affects mainly the downstream genes and proteins expressions involved [78]. Interestingly in metastatic cancer, *OBSCN* mutation observed with new list of interacting genes in Ras protein family members such as *RHOD, RHOT2* and *RAC3* are involved in major functions such as reorganization of actin cytoskeleton, GTPase activity, formation of lamellipodia, respectively [79–81, 99]. The *RAC3* gene is important gene activates *PAK1*, which is vital to stimulates F-actin formation leads to cell migration. Similarly, *PAK1* activates another important proliferation related protein, aurora kinase (*AURKA*) and it inhibits *IKB*-α, which inhibits apoptosis through *NF*-ĸB and *CIAP1* genes [82, 83]. The detailed *OBSCN* gene involved cancer-associated pathway illustrated in Figure 7. The Panther database (http://pantherdb.org/) was used to further validate gene ontology based molecular functions analysis and further confirmed by evaluating fold enrichment scores [84]. The *OBSCN* gene and their networks neighbors were involved in binding and cell regulatory mechanisms including cell adhesion and binding with several receptors and signaling molecules (Figure 8).

### Mutational tolerance profiles and their consequences in proteome level

**Figure 9:**
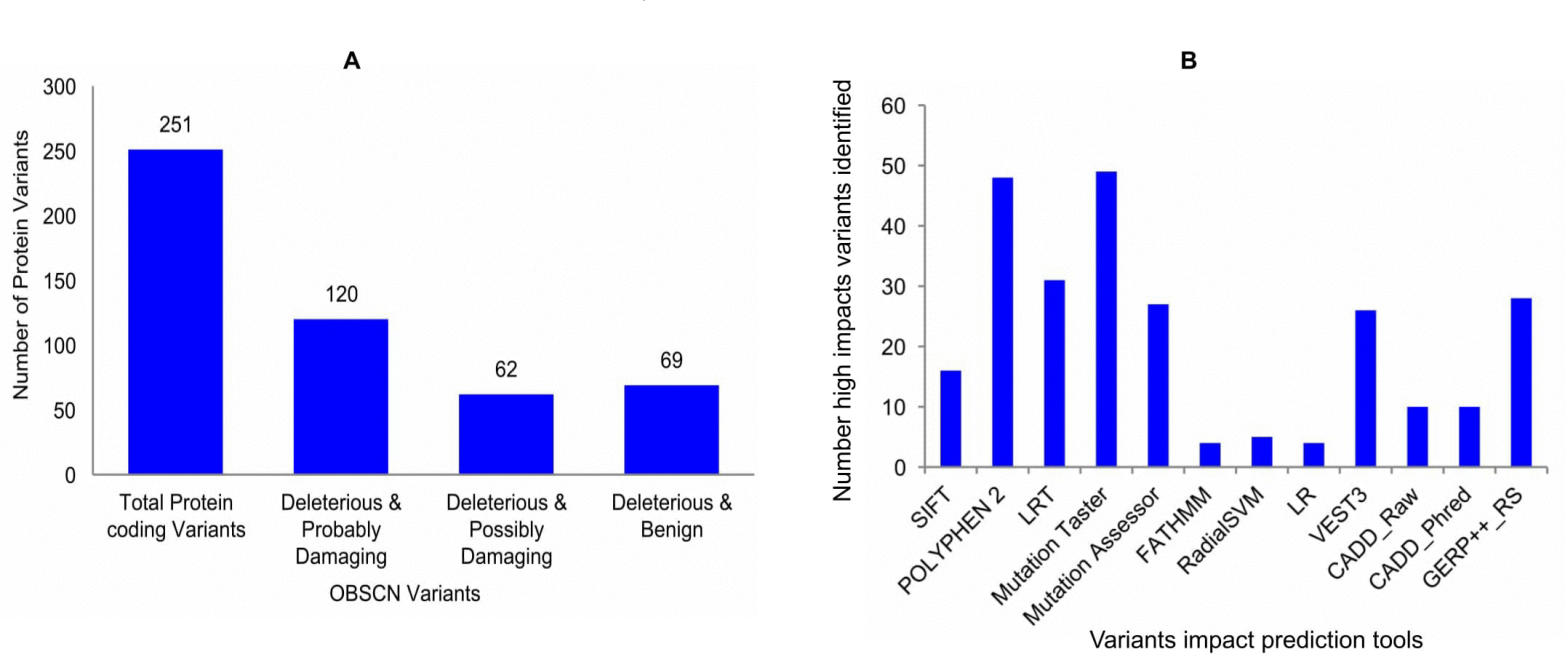
(**A**) SIFT and POLYPHEN-2 programs were used to predict the most common and significant *OBSCN* mutational impact variants; (**B**) To validate the high-impact variants and their mutations impact are predicted using 10 potential impact prediction tools.

**Figure 10:**
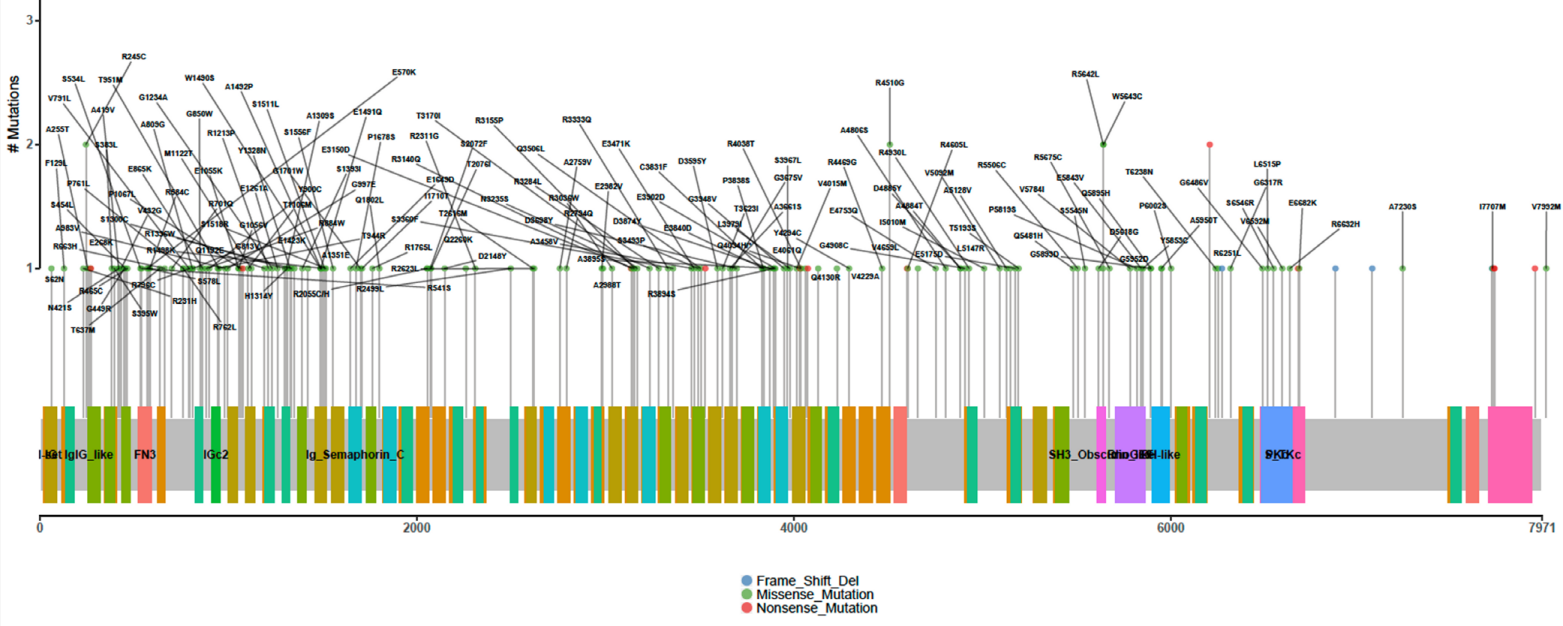
Lollipop plot function of maftools represents functional amino acids mutations and their impacts on various domains of *OBSCN* gene are indicated in different colors representation

Recent studies on *OBSCN* mutations uncover key amino acids mutations, which are crucial factor for many human diseases including cardiomyopathy and cancers. [49, 51, 85]. In this study, we also attempted to know the deep impact of *OBSCN* mutations including the proteome level. The *OBSCN* gene mutational protein variants retrieved from Ensembl genome browser (http://asia.ensembl.org/) and their effects were predicted using Variant Effect Predictor (VEP) tool (http://asia.ensembl.org/Tools/VEP) and wANNOVAR (http://wannovar.wglab.org/) [86, 87]. The SIFT (http://sift.jcvi.org/) and PolyPhen-2 (http://genetics.bwh.harvard.edu/pph2/) tools were used to achieve the most reliable protein variants prediction, and we also considered few other variants impact prediction algorithms such as Log ratio test (LRT), Mutation Taster (http://www.mutationtaster.org/), Mutation Assessor (http://mutationassessor.org/r3/), FATHMM (http://fathmm.biocompute.org.uk/) and Variant effect scoring tool 3 (VEST3)(http://karchinlab.org/apps/appVest.html), etc. Most of the abovementioned tools predict the variants and their impacts based on sequence changes and very few tools also consider structural changes [88–96]. The variants predicted by the maximum number of tools were used to filter and used for further analysis (Figure 9) (http://compbio.berkeley.edu/proj/varant/manual.html). From the overall analysis, it was seen that *OBSCN* gene had a low tolerance against few functional mutations (total of 251 protein variants), half of the variants (~48%) exhibited deleterious and probably damaging effects (Supplementary Table 1), and rest of the variants came in deleterious & possibly damaging and deleterious with benign categories [95, 97, 98]. The overlapping mutational impact variants were taken and further analyzed the somatic mutations using COSMIC database. The detailed *OBSCN* mutational impact on protein levels are emphasized using lollipop plot of maftools (Bioconductor R-package) and amino acids variations and their impacts are indicated in various colors representations along with mutations count (Figure 10) (https://github.com/PoisonAlien/mafTools). In addition, the key mutational variants, types, amino acid variations and impacts in domain levels are listed in Table 2.

**Table 2:** The significant *OBSCN* mutation variants and their impacts filtered from cBioPortal breast cancer patient tumor samples

Genomic Coordinate	Reference and Variants	Mutation Type	Copy Number	Amino acid Variant	Major Domain Affected
228402602	C>G	Missense	AMP	T544S	Fibronectin-III
228412169	NA	Missense	Gain	V888G	Ig-Domain
228432150	T>G	Missense	Gain	V1212G	Ig-Domain
228432150	NA	Missense	Gain	V1120G	Ig-Domain
228432244	G>T	Missense	Gain	Q1243H	Ig-Domain
228434340	C>T	Missense	Diploid	T1382M	Ig-Domain
228461665	G>A	Missense	Gain	V1778M	Fibronectin-III
228464221	G>-	Frameshift DEL	Del	V2282fs	Ig-Domain
228464225	->G	Frameshift INS	Gain	Q2283fs	Ig-Domain
228479785	NA	Missense	Gain	G3509V	Ig-Domain
228479785	G>T	Missense	Gain	G3938V	Ig-Domain
228479785	G>T	Missense	Gain	G3693V	Ig-Domain
228480325	T>C	Missense	Gain	W3998R	Ig-Domain
228480325	T>C	Missense	Gain	W3753R	Ig-Domain
228480325	NA	Missense	Gain	W3569R	Ig-Domain
228481236	C>T	Missense	Gain	R3868C	Ig-Domain
228491624	AC>-	Frameshift DEL	Del	L4419fs	Ig-Domain
228492905	G>C	Missense	Gain	S4538T	Ig-Domain
228494273	G>A	Missense	Gain	E4911K	Ig-Domain
228494273	NA	Missense	Gain	E3954K	Ig-Domain
228494979	NA	Missense	AMP	K4071N	Ig-Domain
228497221	G>A	Missense	Gain	V5282M	Ig-Domain
228497221	NA	Missense	Gain	V4325M	Ig-Domain
228497221	G>A	Missense	Gain	V5037M	Ig-Domain
228503574	G>A	Missense	Gain	E5059Q	Ig-Domain
228506585	T>C	Missense	Gain	V5668A	SH3
228506585	NA	Missense	Gain	V4711A	Ig-Domain
228506585	T>C	Missense	Gain	V5423A	Ig-Domain
228506947	G>A	Missense	Gain	A5544T	NA
228509882	G>A	Missense	Gain	E5826K	NA
228521392	NA	Missense	Gain	S5322C	Ig-Domain
228521466	C>T	Missense	Gain	R6304W	NA
228521466	NA	Missense	Gain	R5347W	Ig-Domain
228521466	C>T	Missense	Gain	R6059W	Ig-Domain
228537694	->T	Frameshift INS	Gain	Y6796fs	NA
228538601	C>T	Nonsense	Gain	Q6838^*^	Ig-Domain
228547542	G>A	Missense	Gain	G6317R	NA
228556454	G>T	Missense	Gain	R7312L	STK-cat Domain
228559643	C>A	Missense	Gain	P7767Q	NA
228559663	C>T	Missense	Gain	P7774S	Protein Kinase 2

^*^Ig- Immunoglobulin. AMP-Amplification; Del-Deletion

### *OBSCN* mediated targeted anti-cancer therapy: a present and future perspectives

Several interesting genes are interlinked with tumorigenesis including *OBSCN* gene, which is considered the key gene among them. The *OBSCN* gene may act as unique target for anti-cancer therapy in breast and other cancers, since it is having multiple interactions with intra and inter-cellular levels of numerous interconnecting proteins. In breast cancer targeted anti-cancer therapy, several key genes are targeted such as *CTNND1*, *CDC42* and *DVL*. Over expression of *P120-CATENIN* tremendously inhibited several signaling molecules such as *RHOA, RAC1* and *CDC42* which are involved in the regulation of numerous cellular functions [100]. Similarly, downregulation of *CDC42* initiated cancer. Interestingly *OBSCN* gene is an active target regulating *CDC42* downstream pathway accounting for the tumorigenesis [101]. DVL (Dishevelled homology1) protein phosphorylation are vital phenomenon accelerated by *CK1ε* and *RIPK4* in Wnt signaling pathway [102]. Hence, reduced β-catenin level and LRP6 association facilitates further downstream signaling of β-catenin, which activates transcription factors/ lymphoid enhancer-binding factors (TCF/LEF) involved in numerous cellular regulations and cell migrations. An alternative and most interesting target is DVL expression responsible for calmodulin-dependent protein kinase (CamK-II) activation followed by expression of F-actin. The F-actin is the key protein involved in cell division, contraction and locomotion and altered F-actin dynamics is most vital feature for epithelial to mesenchymal transition (EMT) of metastatic breast cancer. Hence, anti-cancer drug targets may actively repress DVL and it may potentially block F-actin dynamics, which inhibits EMT process in cancer cells, and the overall phenomena, is indirectly regulated by *OBSCN* gene.

### Concluding remarks

We have analyzed *OBSCN* gene mutation, expression and methylation data and the data revealed that *OBSCN* gene is one of most frequently mutated gene in various cancer types, especially in breast cancer. The *OBSCN* gene mutation may play an essential role during cancer initiation and progression, largely distressed by various kinds of mutations in gene level due to loss of heterozygosity, various oncogenic factors, intra and intercellular environmental stress. Mutations in *OBSCN* gene largely affects multiple properties of cells including cell adhesion and increase the integration and render cellular transitions and many more. Evaluation of *OBSCN* mutational status may help early prognosis of metastatic potential of breast cancer. Mutational, copy number and epigenetic analysis of *OBSCN* gene status may serve as a tool for the prediction of targeted anti-cancer drugs, which will be helpful for targeted breast cancer therapy [11, 20]. Further functional studies on *OBSCN* gene in various model systems with gene overexpression and/or targeted disruption should also greatly facilitate these processes.

## SUPPLEMENTARY MATERIALS FIGURES AND TABLES




