# Supplementary material for: A comprehensive genomic meta-analysis identifies confirmatory role of OBSCN gene in breast tumorigenesis
Source: Oncotarget. 2017 Aug 23;8(60):102263–76. doi: 10.18632/oncotarget.20404 (PMC5731952; doi:10.18632/oncotarget.20404)
Supplement: Supplementary file 1 [file oncotarget-08-102263-s001.pdf]

A comprehensive genomic meta-analysis identifies confirmatory role of *OBSCN* gene in breast tumorigenesis

SUPPLEMENTARY MATERIALS

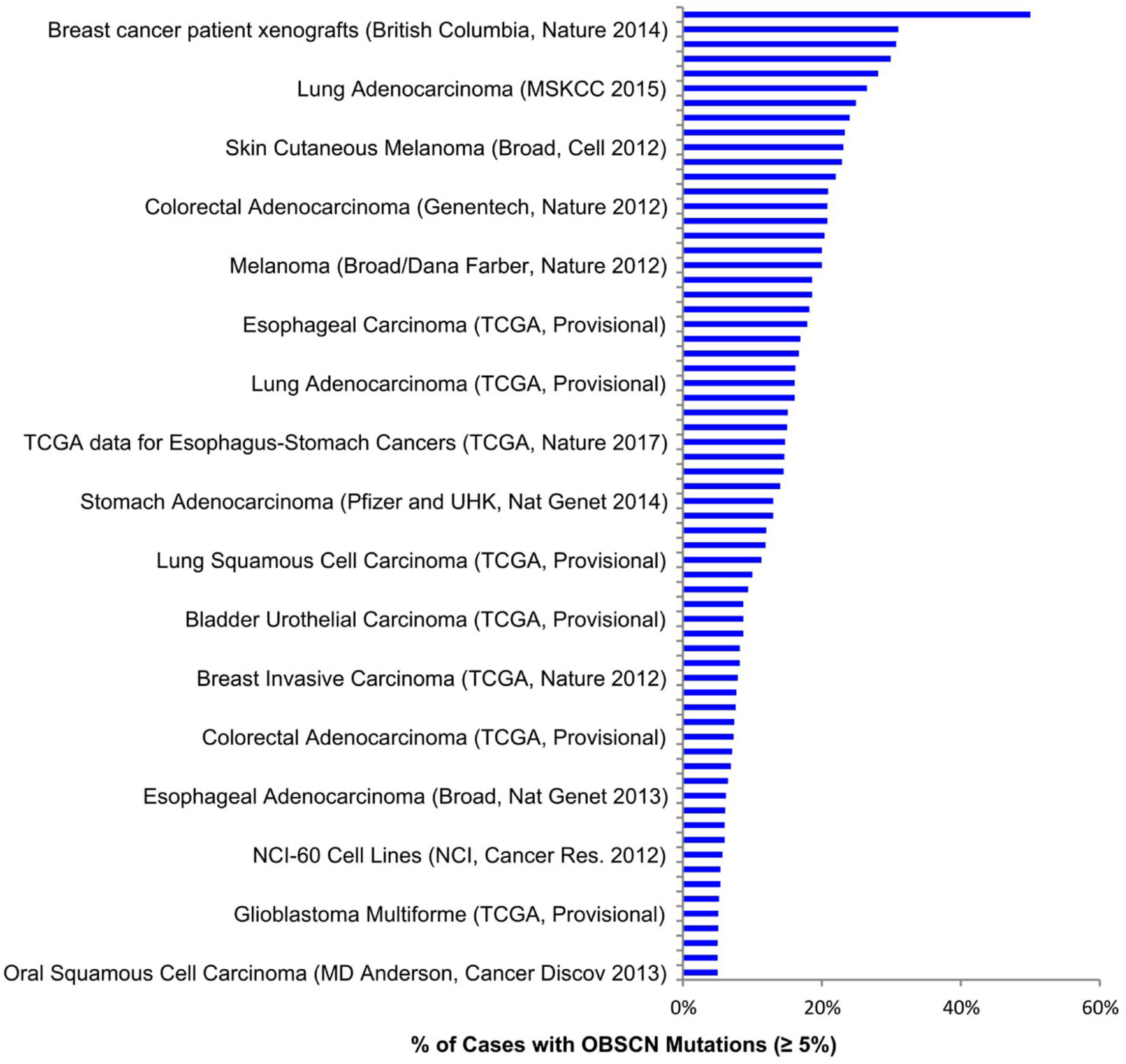

Supplementary Figure 1: Percentage of cases with *OBSCN* gene mutations over various cancer types (> 5%).

Supplementary Table 1: Protein variants of *OBSCN* gene predicted using SIFT and POLYPHEN-2 with reference SNP (rs-id) identifier, gene and protein variants information with SIFT and Polyphen-2 scores. See Supplementary_Table_1
