# Supplementary material for: A comprehensive genomic meta-analysis identifies confirmatory role of OBSCN gene in breast tumorigenesis
Source: Oncotarget. 2017 Aug 23;8(60):102263–76. doi: 10.18632/oncotarget.20404 (PMC5731952; doi:10.18632/oncotarget.20404)
Supplement: Supplementary file 2 [file oncotarget-08-102263-s002.docx]

**Supplementary Table 1:** Protein variants of *OBSCN* gene predicted using SIFT and POLYPHEN-2 with reference SNP (rs-id) identifier, gene and protein variants information with SIFT and Polyphen-2 scores

| **Variation ID** | **Ref. Codon** | **Altered Codon** | **Reference**  **Amino acid Residue** | **Altered Amino acid variants** | **SIFT Score** | **PolyPhen 2 Score** |
| --- | --- | --- | --- | --- | --- | --- |
| rs770836618 | GCC | GAC | A | D | 0 | 0.997 |
| rs754769904 | GCC | CCC | A | P | 0 | 0.997 |
| rs762063089 | GCT | CCT | A | P | 0.01 | 0.956 |
| rs766763176 | GCC | ACC | A | T | 0 | 0.991 |
| rs775749451 | GCC | ACC | A | T | 0 | 0.998 |
| rs770319781 | GCG | ACG | A | T | 0 | 0.985 |
| rs750402842 | GCC | GTC | A | V | 0 | 1 |
| rs764974541 | GCG | GTG | A | V | 0 | 0.995 |
| rs758546572 | TGC | GGC | C | G | 0 | 0.984 |
| rs762615209 | TGT | GGT | C | G | 0 | 0.956 |
| rs375165947 | TGC | CGC | C | R | 0 | 1 |
| rs754680220 | TGT | CGT | C | R | 0 | 0.997 |
| rs774081098 | TGC | TAC | C | Y | 0 | 1 |
| rs865840377 | TGC | TAC | C | Y | 0 | 1 |
| rs368739297 | TGT | TAT | C | Y | 0 | 1 |
| rs757575252 | GAC | AAC | D | N | 0 | 0.964 |
| rs771893141 | GAC | AAC | D | N | 0 | 0.967 |
| rs404545 | GAT | AAT | D | N | 0 | 0.991 |
| rs760984220 | GAT | AAT | D | N | 0.01 | 0.991 |
| rs773554102 | GAC | TAC | D | Y | 0 | 0.966 |
| rs760459221 | GAC | TAC | D | Y | 0 | 0.974 |
| rs770877719 | GAT | TAT | D | Y | 0 | 0.997 |
| rs779156128 | GAG | GAT | E | D | 0.02 | 0.966 |
| rs769704612 | GAG | GGG | E | G | 0 | 0.996 |
| rs140186693 | GAG | GGG | E | G | 0 | 0.979 |
| rs551457881 | GAG | GGG | E | G | 0 | 1 |
| rs750595376 | GAG | AAG | E | K | 0 | 0.927 |
| rs752435654 | GAG | AAG | E | K | 0 | 0.97 |
| rs370234174 | GAG | AAG | E | K | 0 | 0.937 |
| rs367706657 | GAG | AAG | E | K | 0 | 1 |
| rs778210691 | GAG | CAG | E | Q | 0 | 0.915 |
| rs745739601 | TTC | GTC | F | V | 0 | 1 |
| rs759737291 | TTC | GTC | F | V | 0 | 1 |
| rs747374490 | GGA | GCA | G | A | 0 | 0.965 |
| rs761747210 | GGC | GCC | G | A | 0.01 | 0.933 |
| rs780661434 | GGC | GAC | G | D | 0 | 0.981 |
| rs375410104 | GGG | GAG | G | E | 0 | 0.965 |
| rs757897389 | GGC | CGC | G | R | 0 | 1 |
| rs747588194 | GGG | AGG | G | R | 0 | 1 |
| rs375623957 | GGG | CGG | G | R | 0 | 0.965 |
| rs758749499 | GGC | AGC | G | S | 0 | 0.988 |
| rs556474884 | GGC | AGC | G | S | 0 | 0.953 |
| rs774133082 | GGC | AGC | G | S | 0 | 1 |
| rs769508862 | GGC | AGC | G | S | 0.01 | 0.985 |
| rs368928854 | GGT | GTT | G | V | 0 | 1 |
| rs750724031 | CAC | TAC | H | Y | 0.01 | 0.986 |
| rs757869871 | ATT | TTT | I | F | 0 | 0.988 |
| rs748764448 | ATC | AGC | I | S | 0 | 0.976 |
| rs371878526 | ATT | ACT | I | T | 0.03 | 0.967 |
| rs747078519 | ATC | GTC | I | V | 0 | 0.918 |
| rs756720189 | ATC | GTC | I | V | 0.03 | 0.998 |
| rs752718321 | AAG | GAG | K | E | 0 | 0.953 |
| rs771854672 | AAG | GAG | K | E | 0 | 0.992 |
| rs376281341 | AAG | AAT | K | N | 0 | 0.996 |
| rs759083412 | AAG | CAG | K | Q | 0 | 0.978 |
| rs201224119 | AAG | AGG | K | R | 0.02 | 0.919 |
| rs202052558 | CTC | TTC | L | F | 0 | 1 |
| rs751140344 | CTC | TTC | L | F | 0 | 1 |
| rs753004469 | CTC | TTC | L | F | 0 | 1 |
| rs755928557 | CTC | ATC | L | I | 0 | 0.976 |
| rs766468565 | CTG | CCG | L | P | 0 | 0.994 |
| rs761994990 | CTG | CCG | L | P | 0 | 0.998 |
| rs368389773 | CTC | CGC | L | R | 0 | 0.968 |
| rs762136425 | CTG | CGG | L | R | 0 | 0.998 |
| rs747262678 | CTG | GTG | L | V | 0 | 0.967 |
| rs375456294 | AAC | AGC | N | S | 0.01 | 1 |
| rs747944570 | CCC | CGC | P | R | 0.04 | 0.986 |
| rs374417116 | CCA | TCA | P | S | 0.02 | 0.92 |
| rs770652160 | CCC | TCC | P | S | 0 | 0.987 |
| rs375061154 | CCC | TCC | P | S | 0.03 | 0.996 |
| rs566340039 | CCC | TCC | P | S | 0.05 | 0.931 |
| rs575123570 | CCG | TCG | P | S | 0.05 | 0.996 |
| rs763989530 | CGC | TGC | R | C | 0 | 0.979 |
| rs758890771 | CGC | TGC | R | C | 0 | 1 |
| rs766698182 | CGC | TGC | R | C | 0 | 0.944 |
| rs775255330 | CGC | TGC | R | C | 0.02 | 0.959 |
| rs746898810 | CGC | GGC | R | G | 0 | 0.972 |
| rs748139109 | CGG | GGG | R | G | 0 | 0.914 |
| rs189169421 | CGC | CAC | R | H | 0 | 1 |
| rs544522632 | CGC | CAC | R | H | 0 | 0.988 |
| rs780128656 | CGC | CAC | R | H | 0 | 0.997 |
| rs367712031 | CGC | CAC | R | H | 0.01 | 0.924 |
| rs764305067 | CGA | CAA | R | Q | 0 | 0.996 |
| rs113202278 | CGG | CAG | R | Q | 0 | 0.994 |
| rs781057736 | CGG | CAG | R | Q | 0 | 1 |
| rs756895377 | CGC | AGC | R | S | 0.03 | 0.993 |
| rs754399619 | CGG | TGG | R | W | 0 | 0.92 |
| rs370871187 | CGG | TGG | R | W | 0 | 0.999 |
| rs762702571 | CGG | TGG | R | W | 0 | 1 |
| rs868338342 | CGG | TGG | R | W | 0 | 0.975 |
| rs765265986 | CGG | TGG | R | W | 0 | 0.93 |
| rs376053586 | CGG | TGG | R | W | 0 | 0.975 |
| rs569176475 | CGG | TGG | R | W | 0 | 0.924 |
| rs761743683 | CGG | TGG | R | W | 0.01 | 0.969 |
| rs765338702 | CGG | TGG | R | W | 0.02 | 0.963 |
| rs763304976 | TCC | TGC | S | C | 0.04 | 0.917 |
| rs758076006 | TCC | TTC | S | F | 0 | 0.952 |
| rs151181660 | TCT | TTT | S | F | 0 | 1 |
| rs145200728 | TCA | CCA | S | P | 0 | 0.991 |
| rs766303596 | TCT | CCT | S | P | 0 | 0.986 |
| rs371912582 | TCG | TGG | S | W | 0 | 0.999 |
| rs150312546 | ACC | ATC | T | I | 0 | 0.993 |
| rs775901971 | ACG | ATG | T | M | 0 | 0.987 |
| rs777274425 | ACG | ATG | T | M | 0.01 | 0.924 |
| rs749516787 | ACG | AGG | T | R | 0 | 0.999 |
| rs761672125 | ACC | AGC | T | S | 0 | 0.994 |
| rs550118531 | GTG | GGG | V | G | 0 | 0.995 |
| rs369768675 | GTG | TTG | V | L | 0 | 0.983 |
| rs372168665 | GTG | ATG | V | M | 0 | 0.995 |
| rs772880238 | GTG | ATG | V | M | 0 | 0.996 |
| rs747400374 | GTG | ATG | V | M | 0 | 1 |
| rs777404708 | GTG | ATG | V | M | 0 | 1 |
| rs752355643 | GTG | ATG | V | M | 0.02 | 0.989 |
| rs764679750 | GTG | ATG | V | M | 0.02 | 0.962 |
| rs553815670 | TGG | CGG | W | R | 0 | 1 |
| rs533863540 | TAC | TGC | Y | C | 0 | 1 |
| rs780189440 | TAC | TGC | Y | C | 0 | 0.919 |
| rs531649517 | TAT | TGT | Y | C | 0 | 0.986 |
| rs757456642 | TAT | TGT | Y | C | 0 | 1 |
| rs773073943 | TAT | CAT | Y | H | 0 | 1 |
